# Predictive Model for Opioid Use Disorder in Chronic Pain: A Development and Validation Study

**DOI:** 10.3390/biomedicines12092056

**Published:** 2024-09-10

**Authors:** Mónica Escorial, Javier Muriel, César Margarit, Laura Agulló, Thomas Zandonai, Ana Panadero, Domingo Morales, Ana M. Peiró

**Affiliations:** 1 Pharmacogenetics Unit, Clinical Pharmacology Department, Alicante Institute for Health and Biomedical Research (ISABIAL), Diagnostic Center, Gray Building, 5th Floor, Avda Pintor Baeza, 12, 03010 Alicante, Spain; jmuriel@umh.es (J.M.); laura.agulloanton@gmail.com (L.A.); tzandonai@umh.es (T.Z.); apeiro@umh.es (A.M.P.); 2 Department of Pharmacology, Paediatrics and Organic Chemistry, Miguel Hernández University of Elche, 03550 Sant Joan de Alicante, Spain; 3 Pain Unit, Dr. Balmis General University Hospital, ISABIAL, 03010 Alicante, Spain; cmargarit69@gmail.com (C.M.); anotapanaquart@gmail.com (A.P.); 4 Department of Psychology and Cognitive Science, University of Trento, 38060 Trento, Italy; 5 Operations Research Center, University Miguel Hernández de Elche, 03202 Elche, Spain; d.morales@umh.es; 6 Institute of Bioengineering, Miguel Hernández University, 03202 Elche, Spain

**Keywords:** opioid use disorder, predictive model, chronic non-cancer pain, chronic opioid use, ambulatory follow-up, prevention

## Abstract

Background/Objective: There are several questionnaires for the challenge of anticipating opioid use disorder (OUD). However, many are not specific for chronic non-cancer pain (CNCP) or have been developed in the American population, whose sociodemographic factors are very different from the Spanish population, leading to scarce translation into clinical practice. Thus, the aim of this study is to prospectively validate a predictive model for OUD in Spanish patients under long-term opioids. Methods: An innovative two-stage predictive model was developed from retrospective (*n* = 129) and non-overlapping prospective (*n* = 100) cohorts of real-world CNCP outpatients. All subjects used prescribed opioids for 6 or more months. Sociodemographic, clinical and pharmacological covariates were registered. Mu-opioid receptor 1 (*OPRM1*, A118G, rs1799971) and catechol-O-methyltransferase (*COMT*, G472A, rs4680) genetic variants plus cytochrome P450 2D6 (CYP2D6) liver enzyme phenotypes were also analyzed. The model performance and diagnostic accuracy were calculated. Results: The two-stage model comprised risk factors related to OUD (younger age, work disability and high daily opioid dose) and provided new useful information about other risk factors (low quality of life, *OPRM*-G allele and CYP2D6 extreme phenotypes). The validation showed a satisfactory accuracy (70% specificity and 75% sensitivity) for our predictive model with acceptable discrimination and goodness of fit. Conclusions: Our study presents the results of an innovative model for predicting OUD in our setting. After external validation, it could represent a change in the paradigm of opioid treatment, helping clinicians to better identify and manage the risks and reduce the side effects and complications.

## 1. Introduction

Opioid analgesics are recognized as a legitimate medical therapy for selected patients with severe chronic non-cancer pain (CNCP) that does not respond to other therapies [1]. In Spain, the use of opioid analgesics has increased by almost 54% overall from 2013 to 2019. However, opioids are associated with risks that include aberrant drug-related behaviors and opioid use disorder (OUD) in up to 20% of regular opioid prescriptions [2]. OUD is detailed in DSM-5 as an unsuccessful effort to cut down or control use, social problems and a failure to fulfill major role obligations [3]. Thus, clinical tools, greater understanding and better assessment are needed to properly screen and monitor patients under long-term opioid prescriptions. 

There is not enough understanding of the inter-individual variability in analgesic administration and aberrant opioid-related behaviors [4]. The scientific literature has indicated that some genetic polymorphisms (mu-opioid receptor 1-*OPRM1* (A118G, rs1799971), *CYP2D6* (10 single nucleotide polymorphisms and copy number variation) and cathecol-O-methyltransferase-*COMT* (G472A, rs4680), among others) may as well contribute to inter-individual differences in morphine consumption [5,6]. 

In our Pain Unit (PU), a predictive model for OUD [7] was developed from a retrospective cohort of patients under chronic use of opioids [8,9,10,11]. This model included these actionable pharmacogenetic markers and other covariates such as age, employment status and equivalent morphine daily dose (MEDD). In this way, the aim of this study was to internally validate the model, in real-world CNCP outpatients, for future clinical translation. We also examined the characteristics of routinely ambulatory CNCP patients under long-term use of opioids.

## 2. Materials and Methods

### 2.1. Patients

An observational cross-sectional study was conducted on patients who routinely attended the PU of Dr. Balmis General University Hospital. This study was approved on 15 May 2020, by the Institutional Review Board (code PI2020-047) where procedures were carried out in accordance with the Declaration of Helsinki. Enrolment began in September 2021 and ended in July 2022. All patients included were ≥18 years old with CNCP (moderate or severe pain lasting for six or more months) under long-term opioids (≥6 months). The exclusion criteria were oncologic pain, opioid prescription <6 months and prior inclusion in the retrospective cohort (model development). Informed consent was obtained from all subjects involved in this study. This manuscript adheres to the applicable STROBE guidelines. 

### 2.2. Procedure and Variables

All subjects enrolled were attended by the research staff for data and saliva sample collection. The variables were collected, in both groups (retrospective and prospective), at the time of the enrolment. They were collected through patient self-report, validated scales and questionnaires (described below) and completed through Electronic Health Records (EHRs). After data collection, patients followed their routine clinical visit. Here, a medical doctor (anaesthesiologist or clinical pharmacologist expert on pain) assessed the OUD diagnosis based on DSM-5 criteria [12].

Sex (female/male), age, employment status (yes/no: active, retired, with work disability-permanent or temporary, unemployed or homemaker) were registered. The cut-off points for monthly incomes were established according to the Spanish minimum interprofessional wage (EUR 1000) and the minimum vital income (EUR 500) to facilitate the translation to other countries. Thus, data were categorized into low incomes—less than EUR 500, middle incomes—between EUR 500–1000 or upper incomes—more than EUR 1000.

The presence/absence of current and/or previous substance use disorders (SUDs) (except opioid use) related to tobacco, alcohol or other illicit drugs were collected through the review of medical diagnoses, narratives or any visit to the Addictive Behavior Unit.

Pain intensity and relief and quality of life were measured using the Visual Analogue Scale (VAS) [13] included in the Global Pain State questionnaire [14], already validated. The VAS for each indicator consists of a 100 mm horizontal line ranging from 0 (lowest) to 100 mm (highest). Specifically, quality of life was measured with the validated EuroQol-5D-3L scale (registration number: 48802, available at https://euroqol.org/, accessed on 30 May 2021), which also includes a Health Utility Score [15] (five dimensions: mobility, self-care, usual activities, pain/discomfort and anxiety/depression; scores from 0 for death to 1 for perfect health). In addition, the health resources use (any recent emergency department (ED) visit, hospitalization or drug changes due to pain and other causes) was registered. 

All and only prescribed drug use was registered and contrasted with EHRs, which allows for reviewing drug prescriptions. Non-opioid analgesics (i.e., paracetamol and metamizole), non-steroidal anti-inflammatory drugs (NSAIDs), weak (i.e., tramadol and codeine) and strong opioids use (i.e., fentanyl, oxycodone, tapentadol, buprenorphine, morphine, hydromorphone and methadone) and immediate release opioids were registered. Buprenorphine, in our clinical setting, is also used for CNCP patients due to its versatility in administration and its manageable side effects. In different opioid combinations, oral MEDD was estimated using available references [16]. The prescription of antidepressants (i.e., amitriptyline, duloxetine and escitalopram), benzodiazepines and neuromodulators (i.e., pregabalin, lacosamide, gabapentin) were also collected. 

What is more, patients’ reports of adverse events (AEs) were collected through a list with the most frequent adverse drug reactions (ADRs, selected according to opioid Summary of Product Characteristics frequency as “very common” and “common”) [17] and a blank space to collect any other adverse event presented. In addition, patients were asked about any depression or anxiety symptoms. They were also grouped by systems according to the Medical Dictionary for Regulatory Activities Terminology—MedDRA (available at https://www.meddra.org, accessed on 14 June 2022) [18,19].

### 2.3. Pharmacogenetic Analysis

Approximately 2 mL of saliva was collected in tubes containing 6 mL of PBS. Once the saliva sample was taken, it was stored at −80 °C until its processing. Genomic DNA was extracted using E.N.Z.A. forensic DNA kit (Omega Bio-Tek Inc., Norcross, GA, USA) following the manufacturer’s instructions. The following gene variants were genotyped: *OPRM1* (rs1799971, A118G), *COMT* (rs4680, G472A) and CYP2D6*2, *3, *4, *5, *6, *10, *17, *29, *35, *41, xN using the real-time PCR rotor gene Q system (Qiagen, Hilden, Germany), through the use of specific TaqMan MGB^®^ probes (Applied Biosystems, Waltham, MA, USA). Amplification parameters were as follows: pre-PCR section 10 min at 95 °C, 40 cycles for 15 seconds of denaturation at 92 °C and 1-min final extension at 60 °C. The specifications regarding conversion from genotype to phenotype are in Appendix A. 

### 2.4. Statistical Methods

It was expected to have 20 cases and 100 controls from the PU for 11 months based on the inclusion rate of cases in the previous study [8]. We anticipated withdrawals, incomplete data or losses to teleassistance instead of PU visits, shrinking the targeted enrolment to 100 patients.

We compared all the variables between the retrospective cohort (Sample 1) and the new cohort (prospective cohort, Sample 2) using χ^2^ or Fisher’s exact test for categorical variables and t-test or U Mann–Whitney test for continuous variables, depending upon their distribution. Here, data distribution was analyzed with the Kolmogorov–Smirnov test using the Lilliefors correction method. Quantitative parametric data (pain intensity, relief and quality of life) are presented as mean and standard deviation (SD), whilst the median and interquartile range (IQR) were used for not parametric data (age, quality of life—Health Utility Score, MEDD, AEs). Categorical data (sex, employment status, income, prior SUD, health resource use, drug use, AEs and genotypes) are expressed as percentages (%). Gene frequencies were compared using the chi-squared χ^2^ goodness-of-fit test. For the *OPRM1* genotype, the G-carriers (subjects with AG or GG genotypes) were grouped due to the limited G allelic frequency. 

We applied the logistic regression model previously developed from Sample 1 (Table S1) to calculate the risk of each individual as follows: eζ/(1 + eζ), where the linear predictor ζ = b_0_ + b_1_x_1_ + b_2_x_2_ + … + b_p_x_p_ contains five independent risk factors. In other words, ζ = 1.633 − 0.072 age + 2.012 work disability + 0.006 MEDD − 1.424 *OPRM1* genotype (AG/GG) + 0.075 PM CYP2D6 phenotype + 3.172 UM CYP2D6 phenotype. 

The methodology proposed for the fitting and internal validation of the model was as follows: First, the total sample was randomly divided into two parts: 80% (for model fitting) and 20% (for validation). From here, a two-stage model was proposed for the model adjustment due to the differences observed for (1) classifying patients in Sample 1 or 2 and (2) predicting OUD risk in each Sample (1 and 2). Here, variables were selected for the model on the basis of the investigators’ consensus on relevant measurable variables, the results of previous studies [8,9] and the univariate analysis (*p* < 0.05). Three logistic regression models were constructed based on the standards for the model-building process [20]. The model selection followed two criteria: (1) small Akaike information criterion—AIC and (2) significance of the variables. Non-significant variables were considered if their coefficients were interpretable. On the other hand, the validation was performed on the observations not used (20%) in fitting the model. This percentage was considered in order to more accurately estimate the clinical usefulness (sensitivity and specificity). Calibration (Hosmer–Lemeshow goodness-of-fit statistic) and discrimination (C-statistic, area under the receiver operating curve) were measured to assess the model performance. All statistical analyses were carried out using R (Version 3.2.0; the GNU project, Cambridge, MA, USA) and GraphPad Prism (Version 5.0, Dotmatics, Boston, MA, USA).

## 3. Results

### 3.1. Participants and Variables

**Figure 1:**
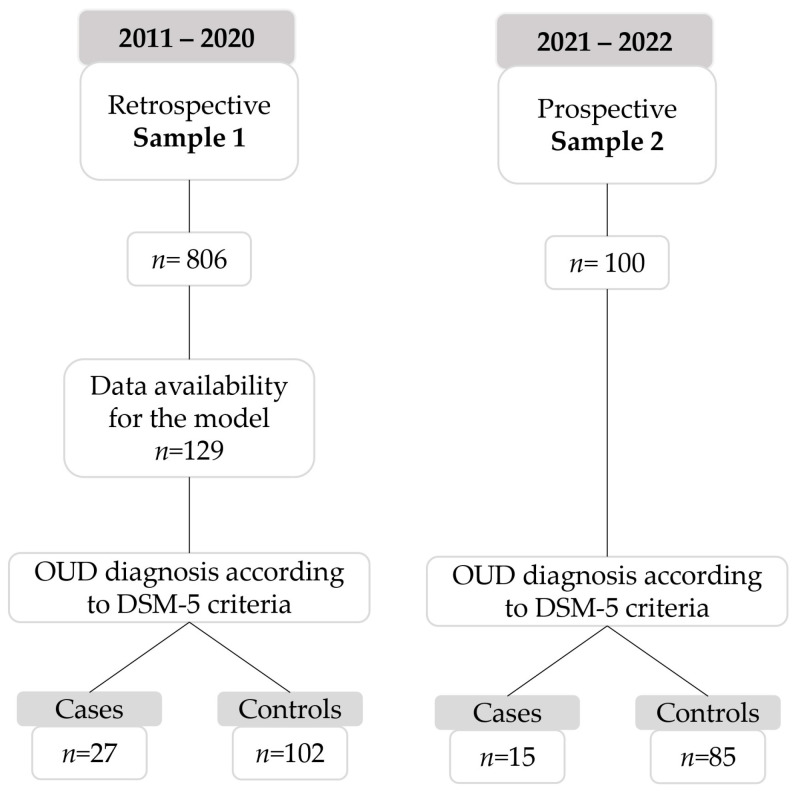
Flow chart of the patients included in a real-world Pain Unit setting.

Sample 1 (*n* = 129) corresponds to the retrospective cohort of patients used originally for the model development (7). Sample 2 (*n* = 100) corresponds to the new prospective cohort of patients recruited, following the detailed inclusion criteria. Three subjects were missing due to insufficient samples for the pharmacogenetic analysis (two for full analysis and one for CYP2D6 analysis) (Figure 1). All patients enrolled attended our PU for regular CNCP management due to lumbalgia (67%, mostly for disc disease pain from spinal canal stenosis with or without a radicular or myofascial pain component), knee pain (gonalgia) and other musculoskeletal pain (i.e., arthralgia and cervical joint dysfunctions).

Middle-aged (63–65 years old), predominantly female (67–70%), retired (50–40%) with 18–25% of a previous SUD (mostly tobacco 71–96%) were the main characteristics of the population included. Half (53–67%) presented middle incomes; however, a higher prevalence of upper incomes (42% vs. 13%, *p* = 0.04) and lower tobacco use (71% vs. 96%, *p* = 0.03) was evidenced in Sample 2, as seen in Table 1. 

Patients of the prospective cohort (Sample 2) suffered higher pain intensity (70 (26) vs. 61 (28) mm, *p* = 0.02) with the near double use of non-opioid analgesics (63% vs. 34%, *p* < 0.001) and tramadol (45% vs. 22%, *p* < 0.001). However, less MEDD (median (IQR), 60 (33–108) vs. 80 (40–160) mg/day, *p* < 0.01) and immediate-release opioids prescription (10% vs. 24%, *p* < 0.01) but higher benzodiazepines use (54% vs. 35%, *p* < 0.01) was observed in front of the retrospective cohort (Sample 1). A different oxycodone, tapentadol and buprenorphine use was also observed between Samples. In addition, the number of AEs reported (2 (1–3) vs. 6 (3–8), *p* < 0.001) were significantly lower in Sample 2 (Table S3). 

### 3.2. Model Performance

Due to the differences observed between Sample 1 and 2, the logistic regression model previously developed from Sample 1 did not present good sensitivity (fewer false negatives, 14%) in the new cohort of patients (Sample 2). In this way, a two-stage model was proposed for the adjustment of the developed model. 

A total of nineteen variables were selected according to the established criteria (see in statistical analysis) and entered in the logistic regression models as candidate predictors: age, employment status (active, work disability and unemployed), prior SUD, pain intensity, quality of life, tramadol use, MEDD, strong opioids use, fentanyl use, benzodiazepines use, ED visits, vomiting, sleep disturbance, psychiatric AEs, *OPRM1* genotype (AA, AG/GG), *COMT* genotype (GG, GA and AA) and CYP2D6 phenotypes (PM, EM and UM) (Table S2)) [9,21,22,23,24,25].

Firstly, a logistic regression model was developed to classify patients in Sample 1 or 2 (Table 2). This model included nine independent factors: ζ = 0.242 − 1.950 active − 1.740 work disability − 3.976 unemployed + 0.004 MEDD + 3.493 strong opioid use − 2.626 benzodiazepines use − 1.496 ED visits + 2.289 psychiatric AEs − 2.159 *COMT* genotype (GA) − 0.901 *COMT* genotype (GG). Here, the cut-off point (c = 0.57) presented the optimal values for specificity (0.86) and sensitivity (0.85). 

Secondly, two logistic regression models were developed to estimate OUD risk in each Sample. The newly developed model for Sample 1 (Table 3) included four independent factors: ζ = −0.622 − 0.057 age + 2.859 work disability + 0.006 MEDD + 1.191 PM CYP2D6 phenotype + 3.299 UM CYP2D6 phenotype. Here, the optimal values of specificity (0.82) and sensitivity (0.94) were obtained with a cut-off point of 0.18. The C-statistic indicated a satisfactory model discrimination (0.86). The model’s ability to accurately predict the likelihood of developing OUD was measured with the test Hosmer–Lemeshow (*p* = 0.26), which indicated a limited model fit.

The predictive model for Sample 2 (Table 4) included three independent factors: ζ = −1.713 − 0.032 quality of life + 0.006 MEDD + 1.017 *OPRM1* genotype (AG/GG) with an optimal cut-off point of 0.19 for satisfactory specificity (0.78) and sensitivity (0.73). The C-statistic indicated a satisfactory model discrimination (0.86). The Hosmer–Lemeshow (*p* = 0.36) showed a limited model fit.

### 3.3. Model Validation

The model developed for classifying patients in Sample 1 or 2 had a satisfactory sensitivity and specificity (0.78 and 0.68, respectively) with a cut-off point of 0.35. On the other hand, the models for predicting OUD risk presented adequate sensitivities (0.75 both Samples) and specificities (0.81 and 0.57, Sample 1 and Sample 2) for a cut-off point of 0.08 and 0.10, respectively. Thus, the two-stage model presented on average 70% specificity and 75% sensitivity.

## 4. Discussion

We have developed and internally validated a predictive model as a screening tool that can classify CNCP patients at risk for OUD when they are under long-term opioids. This tool consisted of a two-stage model that comprised well-documented risk factors related to OUD (younger age, work disability and high MEDD) and provided more useful information about other less-explored risk factors (low quality of life, OPRM-G allele and CYP2D6 extreme phenotypes). 

One of the novel features of this study is the inclusion of genetic variables in the predictive model, which is lacking in many prior tools, including the Opioid Risk Tool. In this era of precision medicine and artificial intelligence, healthcare could benefit from such studies that utilize genetic predictors to stratify patients into risk categories for OUD [26]. What is more, while previous studies have developed predictive models with databases from specific populations (e.g., a veterans’ health administration database) [21,27], our model used regular CNCP patients from ambulatory PU data. 

Clinical guidelines have been established and recommend *CYP2D6* genotype testing prior to prescription of tramadol or codeine, as it has been associated with failure of pain treatment in PM (limited conversion to active metabolites) and a higher risk of AEs in UM [22]. In the case of *OPRM1*, numerous studies have associated the mutant variant (118G) with OUD risk, as they have observed a lower receptor expression in the membrane [23]. In this way, pharmacogenetic testing would allow healthcare providers to individualize prevention strategies [24]

It is well-recognized that MEDD is a major determinant for developing an OUD [25]. Experts have agreed that lower doses of opioids could reduce the risk of opioid use disorder and overdose [3]. Here, daily doses close to or greater than 100 mg/day are at higher risk than dosages < 50 mg/day. Yang S. Liu et al. [28] developed and validated an OUD risk predictive model in 316,039 patients from a national healthcare database, where MEDD was one of the ten top-ranked predictors. Nevertheless, they lacked a clear indicator for treated OUD, and the model interpretability needed further validation. 

Data reveal that the opioid crisis disproportionately impacts some specific populations, such as people with low incomes, with past or current substance abuse and untreated psychiatric disorders [1]. Our data reveal that younger ages with a more vulnerable work status can condition OUD, together with a low quality of life. The latter has been previously linked with SUDs [29]. Thus, recognizing and measuring it in clinical practice should also improve the outcomes in patients with OUD. 

These results need to be interpreted with caution due to their limitations. Firstly, the relatively poor incidence of OUD in our setting, being the result of clinical practice, could have prevented us from detecting the causality and other potential risk factors. Here, it is relevant to mention that patients came from a PU, where the prevalence of OUD is not even described. However, based on the sensitivity and specificity results observed, we could conclude that the predictive capacity of the model is adequate. In this study, we proposed a decision tree, which can be very useful in subpopulations with differences. 

Among other limitations, it is not clear how strong the influence of genetic factors is compared to other factors in predicting OUD. Moreover, the allele frequency of CYP2D6 extreme phenotypes could have limited the detection of OUD risk with more accuracy, although the results showed an adequate sample size. In addition, we should have considered the different metabolism ways related to each opioid drug used and coadjuvants with potential inhibitory or enhancing effects, as they can have different addiction potentials and a different affinity to opioid receptors and therefore different behavioral effects. Prior SUDs were registered from the EHRs, which is limited by the missing information reported by clinicians. However, it has been reported that the prevalence of this variable is lower in prescribed opioid users [30]. Additionally, we only focused on musculoskeletal pain, avoiding other types of pain. In addition, the family history of substance use disorder together with the personality traits could have provided more comprehensive information regarding the risk profile, improving the model accuracy.

Finally, the three genes studied have biologically plausible mechanisms for affecting opioid response (*CYP2D6*—drug metabolism, *OPRM1*—drug target, *COMT*—pain perception). However, additional drug–gene associations have preliminary evidence (ABCB1-morphine, CYP3A4-fentanyl, CYP2B6-methadone) [31] and should be explored in the next study. Here, it is important to highlight that pharmacogenomic screening is not part of routine clinical care yet, but the increasing ease of the availability of genetic data due to the improvement and reduction in the cost of the techniques available for its analysis is progressively helping its implementation [32]. In addition, on 23 June 2023, the National Health Services proposed the inclusion of pharmacogenomic testing in the genetic services. The proposal included a minimum of 12 genes to cover a total of 65 drugs—23 for primary use and 42 for secondary use. In this way, with a blood or saliva sample, we can obtain information for more than 70% of the most common drugs, obtaining a high level of cost-effectiveness. The rest of the variables (clinical and pharmacological) included in the model are easily accessible and captured in clinical care. Thus, it must also be taken into account that there are other factors that influence the variability of the response to drugs, such as some environmental determinants. It is important to standardize the pharmacogenetic report so that it is incorporated together with the rest of the variables. In this way, the implementation of pharmacogenetics must involve multidisciplinary collaboration between health professionals, which allows the assessment of the response to the treatment of each patient taking into account their context, and other clinical variables.

The ultimate purpose of this model is to help identify patients who are prone to develop OUD and who therefore should be closely monitored or may benefit from preventive interventions. This model could only be applied in our center, where it has been developed, and for a certain time. As a future research line for external validation, it is needed to carry out a cross-validation procedure to obtain prediction intervals. At this point, it is crucial to study a generalization of the model in other patient populations and clinical settings, minimizing model overfitting.

## 5. Conclusions

We have developed an innovative predictive model based on real-world data from the Pain Unit routine clinical care in Spain. This model could help to focus on patients requiring monitoring or preventive interventions, optimizing medical resources and improving patients’ quality of life. In this way, external validation is crucial to ensure the clinical usefulness of the model in diverse patient populations and clinical settings. 

## Figures and Tables

**Table 1:** Sociodemographic, clinical and pharmacological characteristics of Samples 1 and 2.

	Retrospective Sample 1 (*n* = 129)	Prospective Sample 2 (*n* = 100)
Sex (% female)	67	70
Age (years old) (median (IQR))	63 (52–72)	65 (52–73)
Employment status (%)		
Active	15	13
Retired	50	40
Work disability	21	26
Unemployed	7	6
Homemaker	7	15
Previous SUD (%)	18	25
Tobacco	96 *	71
Alcohol	4	25
Illicit substances	0	4
Incomes (%)		
Less than EUR 500	20	5
Between EUR 500 to 1000	67	53
More than EUR 1000	13	42 *
Clinical outcomes (mean (SD))		
Pain intensity (VAS, mm)	61 (28)	70 (26) *
Pain relief (VAS, mm)	38 (31)	41 (31)
Quality of life (VAS, mm)	46 (24)	46 (28)
Health Utility (0–1 score) (median (IQR))	0.514 (0.113–0.732)	0.252 (0.051–0.648)
Health resource use (%)		
Emergency room visits	30	42
Hospitalizations	14	25
Medication changes	50	51
Drug prescription (%)		
Non-opioid analgesics	34	63 *
NSAIDs	17	22
Tramadol	22	45 *
MEDD (mg/day) (median (IQR))	80 (40–160) *	60 (33–108)
Oxycodone	67 *	14
Fentanyl	15	24
Tapentadol	11	37 *
Buprenorphine	3	22 *
Morphine	3	3
Hydromorphone	1	0
Immediate release opioids	24 *	10
Neuromodulators	52	60
Antidepressants	50	46
Benzodiazepines	35	54 *

NSAIDs: non-steroidal anti-inflammatory drugs; MEDD: morphine equivalent daily dose, VAS: visual analog scale, SUD: substance use disorder. * *p*-value < 0.05 comparing Sample 1 vs. Sample 2.

**Table 2:** The logistic regression model chosen to classify patients in Sample 1 or 2.

		β-Coefficients	95% CI	Std. Error	z-Value	Pr (>|z|) ^a^
Intercept		0.242	−1.53 to 1.95	0.873	0.277	0.78
Active		−1.950	−3.65 to −0.35	0.831	−2.347	0.02
Work disability		−1.740	−3.05 to −0.54	0.633	−2.750	0.006
Unemployed		−3.976	−5.71 to −2.51	0.809	−4.914	<0.001
MEDD		0.004	−0.00 to 0.01	0.003	1.515	0.13
Strong opioids		3.493	2.05 to 5.24	0.803	4.349	<0.001
Benzodiazepines		−2.626	−3.94 to −1.50	0.617	−4.254	<0.001
ED visits		−1.496	−2.64 to −0.45	0.552	−2.707	0.007
Psychiatric AEs		2.289	1.21 to 3.52	0.583	3.929	<0.001
*COMT*	GA	−2.159	−3.60 to −0.89	0.686	−3.147	0.002
	GG	−0.901	−2.56 to 0.70	0.824	−1.094	0.27

AEs, adverse events, ED: emergency department, MEDD: morphine equivalent daily dose, ^a^ *p*-value associated with the z-value.

**Table 3:** Independent opioid use disorder risk predictors selected in Sample 1.

		β-Coefficients	95% CI	Std. Error	z-Value	Pr (>|z|) ^a^
Intercept		−0.622	−4.77 to 2.99	1.933	−0.322	0.75
Age		−0.057	−0.12 to 0.00	0.032	−1.798	0.07
Work disability		2.860	1.33 to 4.78	0.848	3.373	<0.001
MEDD		0.006	0.00 to 0.01	0.003	2.444	0.02
CYP2D6	PM	1.191	−2.19 to 3.90	1.442	0.826	0.41
	UM	3.299	0.82 to 5.97	1.255	2.628	0.009

MEDD: morphine equivalent daily dose, PM: poor metabolizer; UM: ultra-rapid metabolizer. ^a^ *p*-value associated with the z-value.

**Table 4:** Independent opioid use disorder risk predictors selected in Sample 2.

	β-Coefficients	95% CI	Std. Error	z-Value	Pr (>|z|) ^a^
Intercept	−1.713	−3.35 to −0.31	0.759	−2.256	0.02
Quality of life	−0.032	−0.06 to −0.01	0.014	−2.302	0.02
MEDD	0.005	−0.00 to 0.01	0.004	1.394	0.16
*OPRM1* (AG/GG)	1.017	−0.36 to 2.56	0.727	1.400	0.16

MEDD: morphine equivalent daily dose, ^a^ *p*-value associated with the z-value.

## Data Availability

The data that support the findings of this study are available from the corresponding author upon reasonable request.

## Supplementary Materials

The following supporting information can be downloaded at: https://www.mdpi.com/article/10.3390/biomedicines12092056/s1, Supplementary Data: *CYP2D6*-Conversion; Table S1: Original model developed from Sample 1; Table S2: Justification for the inclusion of specific predictors in the model; Table S3: Safety characteristics of Samples 1 and 2.

## Appendix A

Regarding the CYP2D6 genotype, genetic analysis was based on the usual PCR methods following the instructions of Consortium of the Pharmacogenetics and Pharmacogenomics Ibero-American network 21. These amplifications were carried out in a Mastercycler 384 (Eppendorf, Hamburg, Germany).

The combination of *CYP2D6* alleles is used to determine a patient’s diplotype. Each allele is assigned an activity score (AS) ranging from 0 to 1 (e.g., 0 for no function, 0.25 or 0.5 for decreased function and 1 for normal function) 22. Alleles *3, *4, *5, *6 (AS = 0) have null function, while *10 (AS = 0.25) and *17, *29, *41 (AS = 0.5) a reduced function. Alleles *1, *2, *35 (AS = 1) and duplications *1xN, *2xN, *35xN (AS = 2) have normal and greater function, respectively. If an allele contains multiple copies of a functional gene, the value is multiplied by the number of copies present. Thus, the CYP2D6 activity score is the sum of the values assigned to each allele. The CYP2D6 activity score can be translated into a standardized phenotype classification: null function (poor metabolizer, PM), normal function (extensive metabolizer, EM) and increased function (ultra-rapid metabolizers, UM) [22].
