# Supplementary material for: Predictive Model for Opioid Use Disorder in Chronic Pain: A Development and Validation Study
Source: Biomedicines. 2024 Sep 10;12(9):2056. doi: 10.3390/biomedicines12092056 (PMC11428664; doi:10.3390/biomedicines12092056)
Supplement: Supplementary file 1 [file biomedicines-12-02056-s001.zip › biomedicines-3197445-supplementary.pdf]

Supplementary Data

Tables

Table S1. Original model developed from Sample 1.

		β-coefficients	95% CI	Std. Error	z-value	Pr (>|z|)[a]
Intercept		1.633	-1.32 to 4.63	1.489	1.097	0.27
Age		-0.072	-0.13 to -0.03	0.025	-2.884	0.004
Work disability		2.012	0.86 to 3.25	0.604	3.331	0.001
MEDD		0.006	0.00 to 0.01	0.002	2.633	0.008
OPRM1 (AG/GG)		-1.424	-2.90 to 0.17	0.684	-2.083	0.04
CYP2D6	PM	0.075	-3.21 to 2.56	1.375	0.054	0.96
	UM	3.172	1.33 to 5.23	0.972	3.265	0.001

MEDD: morphine equivalent daily dose, PM: poor metabolizer, UM: ultra-rapid metabolizer
[a]*p-value associated with the z-value.*

Table S2. Justification for the inclusion of specific predictors in the model.

Variable	Selection Criteria
Socio-demographic	
Age	p<0.05 in Sample 1
Active	p<0.05 in Sample 1
Work disability	p<0.05 in Sample 1
Unemployed	p<0.05 in Sample 2
ED visit	Investigators' consensus and previous results [21]
Prior SUD	p<0.05 in Sample 1
Clinical	
Pain Intensity	p<0.05 in Sample 2
Quality of life	p<0.05 in Sample 2
Psychiatric AEs	Investigators' consensus and previous results [22]
Vomiting	Investigators' consensus
Sleep disturbance	p<0.05 in Sample 1
Pharmacological	
Tramadol	p<0.05 in Sample 1
Strong Opioids	p<0.05 in Sample 1
MEDD	p<0.05 in both Samples
Fentanyl	p<0.05 in both Samples

Benzodiazepines	Investigators' consensus and previous results [23]
Genetic	
OPRM1 Genotype	Investigators' consensus and previous results [9, 24]
COMT Genotype	Investigators' consensus and previous results [9, 24]
CYP2D6 Phenotype	Investigators' consensus and previous results [25]

AEs: adverse events, ED: emergency department visits due to pain and other causes, MEDD: morphine equivalent daily dose, SUD: substance use disorder.

Table S3. Safety characteristics of Samples 1 and 2.

	Retrospective **Sample 1** (*n*=129)	Prospective **Sample 2** (*n*=100)
(%)		
Adverse Events (median (IQR))	6 (3 – 8)*	2 (1 – 3)
Sleepiness	55*	14
Dizziness	32*	8
Nausea	22*	9
Vomiting	11*	3
Constipation	63*	38
Itching	22	15
Sexual dysfunction	11*	3
Loss of libido	25*	10
Weight change	40*	7
Headache	36*	18
Skin redness	24*	3
Dry skin	42*	10
Dry mouth	65*	39
Edema	17*	6
Depression	33*	9
Sleep disturbance	39*	26
Nervousness	47*	16
Loss of appetite	29*	7

**p-value<0.05 comparing sample 1. vs. sample 2*
